# Mutational signatures and heterogeneous host response revealed via large-scale characterization of SARS-CoV-2 genomic diversity

**DOI:** 10.1016/j.isci.2021.102116

**Published:** 2021-01-28

**Authors:** Alex Graudenzi, Davide Maspero, Fabrizio Angaroni, Rocco Piazza, Daniele Ramazzotti

**Affiliations:** 1 Inst. of Molecular Bioimaging and Physiology, Consiglio Nazionale delle Ricerche (IBFM-CNR), Segrate, Milan, Italy; 2 Bicocca Bioinformatics, Biostatistics and Bioimaging Centre – B4, Milan, Italy; 3 Department of Informatics, Systems and Communication, Univ. of Milan-Bicocca, Milan, Italy; 4 Department of Medicine and Surgery, Univ. of Milan-Bicocca, Monza, Italy

**Keywords:** Genetics, Phylogenetics, Bioinformatics

## Abstract

To dissect the mechanisms underlying the inflation of variants in the Severe Acute Respiratory Syndrome CoronaVirus 2 (SARS-CoV-2) genome, we present a large-scale analysis of intra-host genomic diversity, which reveals that most samples exhibit heterogeneous genomic architectures, due to the interplay between host-related mutational processes and transmission dynamics. The decomposition of minor variants profiles unveils three non-overlapping mutational signatures related to nucleotide substitutions and likely ruled by APOlipoprotein B Editing Complex (APOBEC), Reactive Oxygen Species (ROS), and Adenosine Deaminase Acting on RNA (ADAR), highlighting heterogeneous host responses to SARS-CoV-2 infections. A corrected-for-signatures *dN/dS* analysis demonstrates that such mutational processes are affected by purifying selection, with important exceptions. In fact, several mutations appear to transit toward clonality, defining new clonal genotypes that increase the overall genomic diversity. Furthermore, the phylogenomic analysis shows the presence of homoplasies and supports the hypothesis of transmission of minor variants. This study paves the way for the integrated analysis of intra-host genomic diversity and clinical outcomes of SARS-CoV-2 infections.

## Highlights

• The intra-host genomic diversity of SARS-CoV-2 samples reveals host-related processes
• Three non-overlapping mutational signatures are inferred from minor variant profiles
• Most mutations are purified, yet many variants exhibit wide frequency spectra
• The study of homoplasies shows that minor variants are transmitted across hosts

## Graphical Abstract

**Figure:**
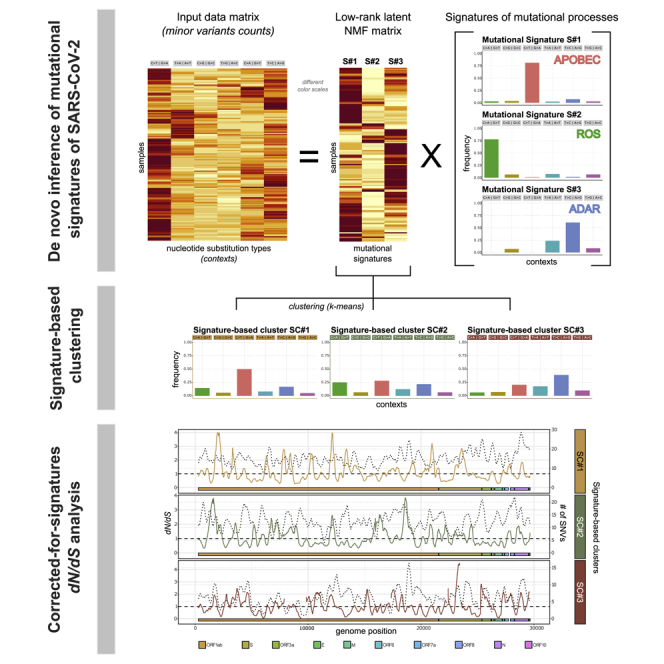

## Introduction

The COronaVIrus Disease 2019 (COVID-19) pandemic has currently affected 216 countries and territories worldwide with ≈70 million people being infected, while the number of casualties has reached the impressive number of ≈1.6 million (World Health Organization (WHO) (2020), update 15^th^ December 2020). The origin and the main features of Severe Acute Respiratory Syndrome CoronaVirus 2 (SARS-CoV-2) evolution have been investigated (Zhou et al., 2020b; Wu et al., 2020; Andersen et al., 2020; Xiao et al., 2020; Deng et al., 2020), also due to the impressive amount of consensus viral sequences included in public databases, such as Global Initiative on Sharing Avian Influenza Data (GISAID) (Shu and McCauley, 2017). However, only a few currently available data sets include raw sequencing data, which are necessary to quantify intra-host genomic variability.

Due to the combination of high error and replication rates of viral polymerase, subpopulations of viruses with distinct genotypes, also known as viral quasispecies (Domingo et al., 1985), usually coexist within single hosts. Such heterogeneous mixtures are supposed to underlie most of the adaptive potential of RNA viruses to internal and external selection phenomena, which are related, e.g., to the interaction with the host's immune system or to the response to antiviral agents. For instance, it was hypothesized that intra-host heterogeneity may be correlated with prognosis and clinical outcome (Novella et al., 1995; Domingo et al., 2012). Furthermore, even if the modes of transmission of intra-host variants in the population are still elusive, one may hypothesize that, in certain circumstances, infections allow such variants to spread, sometimes inducing significant changes in their frequency (Lythgoe et al., 2020).

In particular, several studies on SARS-CoV-2 support the presence of intra-host genomic diversity in clinical samples and primary isolates (Ramazzotti et al., 2021; Shen et al., 2020; Wölfel et al., 2020; Capobianchi et al., 2020; Rose et al., 2020; Lu et al., 2020; Lythgoe et al., 2020; Seemann et al., 2020; Popa et al., 2020), whereas similar results were obtained on Severe Acute Respiratory Syndrome CoronaVirus (SARS-CoV) (Xu et al., 2004), Middle East Respiratory Syndrome (MERS) (Park et al., 2016), Ebola virus (Ni et al., 2016), and Hemagglutinin Type 1 and Neuraminidase Type 1 (H1N1) influenza (Poon et al., 2016). We here present one of the the largest up-to-date studies on intra-host genomic diversity of SARS-CoV-2, based on a large data set including 1133 high-quality samples for which raw sequencing data are available (NCBI BioProject: PRJNA645906). The results were validated on 4 independent data sets including a total of 953 samples (NCBI BioProject:PRJNA625551, PRJNA633948, PRJNA636748, and PRJNA647529; see the Validation section).

Our analysis shows that ≈15% of the SARS-CoV-2 genome has already mutated in at least one sample, including ≈1% of positions exhibiting multiple mutations. The large majority of samples shows a heterogeneous intra-host genomic composition, with 892 out of 1133 samples (≈79%) exhibiting at least one low frequency variant (Variant Frequency, VF >5% and ≤90%, named minor variants or iSNVs), 171 samples more than 5, and 101 samples more than 10. Importantly, several variants are observed as clonal (VF >90%) in certain samples and at a low frequency in others, demonstrating that transition to clonality might be due not only to functional selection shifts but also to complex transmission dynamics involving bottlenecks and founder effects (Domingo et al., 2012).

Strikingly, our analysis allowed us to identify three non-overlapping “mutational signatures”, i.e., specific distributions of nucleotide substitutions, in which are observed in distinct mixtures and with significantly different intensity in three well-separated clusters of samples, suggesting the presence of host-related mutational processes. One might hypothesize that such processes are related to the interaction of the virus with the host's immune system and might pave the way for a better understanding of the molecular mechanisms underlying different clinical outcomes.

In particular, the first signature is dominated by C>T:G>A substitution and it is likely related to APOlipoprotein B Editing Complex (APOBEC) activity, the second signature is mostly characterized by G>T:C>A substitution and it might be associated to Reactive Oxygen Species (ROS)-related processes, while a third signature is predominantly associated to A>G:T>C substitution, which is usually imputed to Adenosine Deaminase Acting on RNA (ADAR) activity.

A corrected-for-signatures version of the dN/dS analysis would suggest that, as expected, the three signatures are affected by mild purifying selection in the population, yet with some exceptions that would suggest the existence of positively selected genomic regions. Furthermore, a certain proportion of samples of two signature-based clusters mostly associated to APOBEC and ROS appear to be hypermutated (up to 87 minor variants detected in a single host), whereas this effect is mitigated for the remaining cluster, dominated by ADAR-related processes.

Finally, the analysis of the phylogenetic model, obtained from the profiles of clonal variants via the Viral Evolution ReconStructiOn (VERSO) framework (Ramazzotti et al., 2021), allowed us to assess how many minor variants are either detected in single samples, in multiple samples of the same clade, or in multiple samples of independent clades (i.e., homoplasies). Strikingly, an approximately monotonic decrease of the median VF is observed with respect to the number of clades in which minor variants are observed: minor variants detected in single clades exhibit the largest (median) VF, as opposed to variants shared in multiple clades, which display a progressively lower VF.

On the one hand, this result supports the hypothesis of transmission of minor variants during infections and of the concurrent existence of bottleneck effects (Gutierrez et al., 2012; Domingo et al., 2012). On the other hand, the significant number of minor variants observed at a low frequency in multiple clades would suggest the presence of mutational hotspots and of phantom mutations related to sequencing artifacts (Bandelt et al., 2002).

## Results

### Mutational landscape of SARS-CoV-2 from variant frequency profiles of 1133 samples – data set #1

We performed variant calling from Amplicon raw sequencing data of 1188 samples from the NCBI BioProject: PRJNA645906 and by aligning sequences to reference genome SARS-CoV-2-ANC, which is a likely ancestral SARS-CoV-2 genome (Ramazzotti et al., 2021). The mutational profiles of 1133 high-quality samples selected after quality check were analyzed in depth (see Methods and Figure S1).

**Figure 1:**
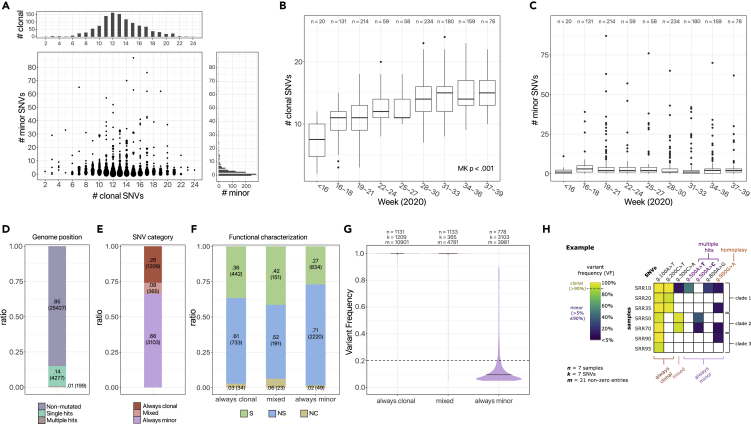
Mutational landscape of 1133 SARS-CoV-2 samples – data set #1 (NCBI BioProject: PRJNA645906) (A) Scatter plot displaying the number of clonal (VF >90%) and minor (VF >5% and ≤90% ) variants for 1133 samples of data set #1 (node size proportional to the number of samples). (B) Box plots returning the distribution of the number of clonal and (C) minor variants, obtained by grouping samples according to collection date (weeks, 2020; Mann-Kendall trend test p value also shown). *n* returns the number of samples in each group. (D) Bar plot returning the proportion of sites of the SARS-CoV-2 genome that are either non-mutated, mutated with a unique SNV, or mutated with multiple SNVs. (E) Stacked bar plots returning the proportion of SNVs detected as always clonal, mixed, or always minor. (F) The ratio of synonymous (S), non-synonymous (NS), and non-coding (NC) mutations, for each category. (G) Violin plots returning the distribution of VF of all SNVs (*n* returns the number of samples, *k* the number of distinct SNVs, *m* the number of non-zero entries of the VF matrix). (H) Graphical representation of an example data set.

In detail, 4677 distinct single-nucleotide variants (SNVs, identified by genome location and nucleotide substitution) were detected in the data set, for a total of 19663 non-zero entries of the VF matrix (see Methods for further details; the VF profiles of all samples are included in Data S1; see Figure 1H for a graphical representation of an example data set). In particular, in our analysis, we consider any SNV detected in any given sample as “clonal”, if its VF is >90% and as “minor” if its VF is >5% and ≤90%.

The distribution of the number of minor and clonal variants observed in each sample (Figure 1A) unveils an approximately normal distribution of clonal variants (median = 13, mean = 13.2, and max = 24). Minor variants are detected in ≈78.7% of the samples and show a long-tail distribution (median = 2, mean = 4.16 and max = 87). One hundred nine samples (≈9.6% of the data set) display a number of minor variants ≥10, up to a maximum of 87.

Interestingly, we observe a statistically significant increase of genomic diversity on clonal variants with respect to collection week (Mann-Kendall test for trend on median number of clonal variants p<0.001, Figure 1B), due to the accumulation of clonal variants in the population, and which confirms recent findings (Li et al., 2020; Shen et al., 2020; Ramazzotti et al., 2021), whereas, as expected, this phenomenon is less evident for minor variants (Figure 1C). This aspect is further investigated in the following and hints at the interplay involving the evolutionary dynamics within hosts and the transmission among hosts, which differently affects clonal and minor variants (Chan et al., 2013).

### Evidence of transition to clonality

We further categorize each detected SNV as follows: (i) “always clonal”, if clonal in all samples in which it is detected, (ii) “always minor”, if minor in all samples in which it is detected, (iii) “mixed”, if observed as clonal in at least one sample and as minor in at least another sample.

Forty thousand six hundred seventy seven SNVs were detected on 4476 distinct genome sites (≈14.9% of the SARS-CoV-2 genome), of which 199 sites (≈0.6% of the genome) display multiple nucleotide substitutions (see Figure 1D). This suggests that the proportion of mutated genomic sites might be considerably higher in the overall population, especially if considering minor variants. Overall, 25.8%, 7.8%, and 66.3% SNVs are detected as always clonal, mixed, and always minor, respectively, and are mostly non-synonymous (see Figures 1E and 1F).

The analysis of the VF distribution (Figure 1G) unveils an impressive scarcity of variants showing VF in the middle range, i.e., between 20% and 90%, for all categories. This phenomenon is likely due to transmission bottlenecks, which tend to purify low-frequency variants in the population. Nonetheless, both mixed and always minor variants display broad VF spectra, an aspect that is particularly relevant for the former category. In this respect, 24.4% of all mixed variants (89 on 365) never display a VF ≤20%: one may hypothesize that such variants are indeed “transiting to clonality” in the population because either positively selected, as a result of the strong immunologic pressure within human hosts (Lucas et al., 2001), or because affected by transmission phenomena involving founder effects, bottlenecks, and stochastic fluctuations (Gutierrez et al., 2012; Domingo et al., 2012).

Conversely, one might hypothesize that most remaining mixed variants may result from random mutations hitting positions of SNVs that are already present as clonal in the population.

**Figure 2:**
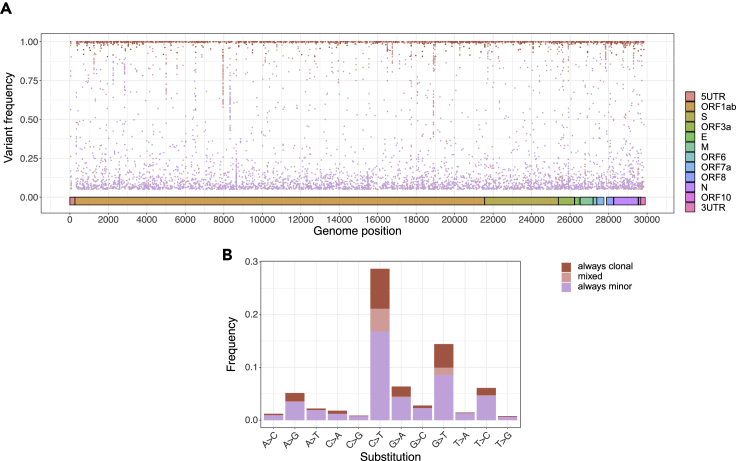
Characterization of SNVs detected on the SARS-CoV-2 genome (A) Scatter plot returning the genome location and the VF of all SNVs detected in the data set, colored according to category. (B) Stacked bar plots returning the normalized substitution proportion of all SNVs detected in at least one sample of the data set, with respect to all 12 possible nucleotide substitutions, grouped by variant type.

Furthermore, the distribution of SNVs with respect to each region of the genome in Figure 2A demonstrates that mutations are approximately uniformly distributed across the genome (also see Figure S2). Overall, this analysis provides one of the first large-scale quantifications of transition to clonality in SARS-CoV-2 and might serve to intercept variants possibly involved in functional modifications, bottlenecks, or founder effects.

### De novo inference of SARS-CoV-2 mutational signatures

In order to investigate the existence of mutational processes related to the interaction between the host and the SARS-CoV-2, we analyzed the distribution of nucleotide substitutions for all SNVs detected in the data set. In Figure 2B, one can see the proportion of SNVs for each of the 12 nucleotide substitution types (e.g., number of C>T's) over the total number of nucleotides present in the reference genome for each substitution type (e.g., number of C's).

Certain substitutions present a significantly higher normalized abundance, confirming recent findings on distinct cohorts (Simmonds, 2020; Di Giorgio et al., 2020; Popa et al., 2020). In particular, C>T substitutions are observed in ≈28% of all C nucleotides in the SARS-CoV-2 genome, G>T's in ≈15% of all G's, T>C's in ≈7% of all T's, and A>G's in ≈5% of all A's.

Although, traditionally, a 12-substitution pattern has been used in order to report mutations occurring in single-stranded genomes, we reasoned that, owing to the intrinsically double-stranded nature of the viral life cycle (i.e., a mutation occurring on a plus strand can be transferred on the minus strand by RdRP and vice versa), it is sound to consider a total of 6 substitution classes (obtained by merging equivalent substitutions in complementary strands) to investigate the possible presence of viral mutational signatures (Alexandrov et al., 2013). Clonal variants were not considered in the next analyses to focus on SNVs likely related to host-specific mutational processes and by excluding variants presumably transmitted during infection events.

In particular, in order to identify and characterize the mutational processes underlying the emergence of SARS-CoV-2 variants with a statistically grounded approach, we applied a Non-negative Matrix Factorization (NMF) approach (Brunet et al., 2004) and standard metrics to determine the optimal rank (see Methods). In particular, we analyzed the mutational profiles of 150 samples exhibiting at least 6 always minor variants (on 1133 total samples) to ensure a sufficient sampling of the distributions.

**Figure 3:**
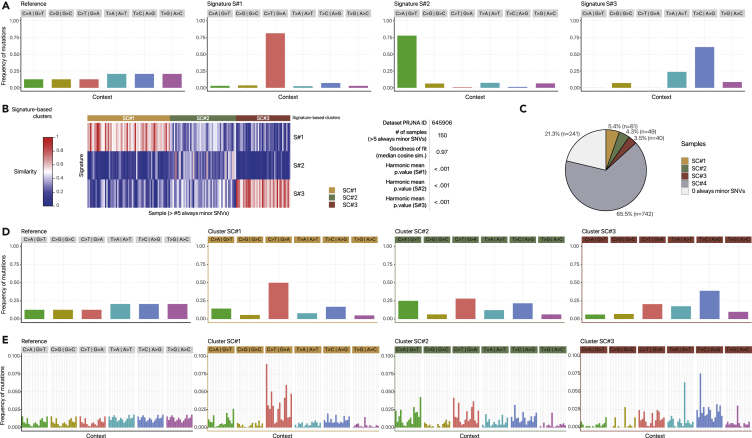
Mutational signatures of SARS-CoV-2 (A) The nucleotide class distribution in SARS-CoV-2-ANC reference genome (Ramazzotti et al., 2021) and for the 3 SARS-CoV-2 mutational signatures retrieved via NMF on 6 substitution classes is shown. (B) Heatmap returning the clustering of 150 samples with ≥6 always minor variants (≈13% of the data set), computed via k-means on the low-rank latent NMF matrix. The goodness of fit in terms of median cosine similarity between observations and predictions and the harmonic mean p value of the one-sided Mann-Whitney U test on bootstrap re-sampling, are shown for all signatures, see Methods. (C) Pie chart returning the proportion of samples in the three signature-based clusters, plus a fourth cluster SC#4 including all samples with ≥1 and <6 always minor variants and the group of samples with 0 always minor SNVs. (D-E) Categorical normalized cumulative VF distribution of all SNVs detected in each signature-based cluster, with respect to (D) 6 substitution classes and to (E) 96 trinucleotide contexts, as compared to the theoretical distribution in SARS-CoV-2-ANC reference genome (left).

Strikingly, 3 distinct and non-overlapping mutational signatures are found and explain 96.5% of the variance in the data (Figures 3A and S4; cophenetic correlation coefficient =0.998, cosine similarity between predictions and observations =0.973, harmonic mean p value of the one-sided Mann-Whitney U test on bootstrap re-sampling <0.01 for all signatures, see Methods). In particular, signature S#1 is predominantly related to substitution C>T:G>A (81.2%), signature S#2 to substitution C>A:G>T (77.7%), while signature S#3 is dominated by substitutions T>C:A>G (S#3) and T>A:A>T (23.6%).

### Characterization of mutational signatures of SARS-CoV-2

Signature S#1 is related to C>T:G>A substitution, which was often associated to APOBEC i.e., a cytidine deaminase involved in the inhibition of several viruses and retrotransposons (Sharma et al., 2015). An insurgence of APOBEC-related mutations was observed in other coronaviruses shortly after spillover (Woo et al., 2007), and it was recently hypothesized that APOBEC-like editing processes might have a role in the response of the host to SARS-CoV-2 (Simmonds, 2020).

As specified above, a mutational process occurring on single-stranded RNA with a given pattern, e.g., C>T, could occur as a C>T mutation on the plus reference strand but could similarly occur on the minus strand, again as a C>T substitution. However, C>T events originally occurring in the minus strand would be recorded as G>A owing to the mapping of the mutational event as a reverse complement on the plus reference genome. Starting from these considerations and hypothesizing that the C>T:G>A substitution is mediated by APOBEC, which operates on single-stranded RNA and is similarly active on both strands, the analysis of the C>T/G>A ratio (or, more generally, of a plus/minus substitution ratio) should give an accurate measurement of the molar ratio between the two viral strands inside the infected cells.

In our case, by comparing the proportion of substitutions of all minor variants detected in the data set, the ratio C>T/G>A is 5.1 $\left(\frac{1602/5491}{335/5863}\right)$.

This result allows us to hypothesize that plus and minus viral strands of the SARS-CoV-2 genome are present in infected cells with a molar ratio in strong favor of the plus strand and are consistent with the expected activity of APOBEC on single-stranded RNA. Further experimental analyses will be required to confirm this hypothesis.

The second signature SC#2 is predominantly characterized by substitution C>A:G>T, whose origin is however still obscure. To gain insight into the mechanisms responsible for its onset, also in this case, we analyzed the C>A and G>T substitution frequency, which revealed a strong disproportion in favor of the latter: the ratio G>T/C>A is 9.5 $\left(\frac{804/5863}{79/5491}\right)$. Overall, this result suggests that, in this case, the G>T substitution is the active mutational process.

In this respect, one might hypothesize a role for ROS as a mutagenic agent underlying this signature, as observed, for instance, in clonal cancer evolution (Alexandrov et al., 2020). ROSs are extremely reactive species formed by the partial reduction of oxygen. A large number of ROS-mediated DNA modifications have already been identified; in particular, however, guanine is extremely vulnerable to ROS because of its low redox potential (David et al., 2007). ROS activity on guanine causes its oxidation to 7,8-dihydro-8-oxo-$2^{\text{′}}$-deoxyguanine (oxoguanine). Notably, (i) oxoguanine can pair with adenine, ultimately causing G>T transversions, and (ii) ROSs are able to operate on single-stranded RNA; therefore, their mutational process closely resembles the C>A:G>T pattern we see in signature SC#2. Thus, it is sound to hypothesize that the C>A:G>T substitution is generated by ROS, whose production is triggered upon infection, in line with several reports indicating that a strong ROS burst is often triggered during the early phases of several viral infections (Molteni et al., 2014; Reshi et al., 2014).

Finally, signature SC#3 is primarily characterized by A>G:T>C substitution, which is typically imputed to the ADAR deaminase mutational process (Nishikura, 2010). ADAR targets adenosine nucleotides, causing deamination of the adenine to inosine, which is structurally similar to guanine, ultimately leading to an A>G substitution. Unlike APOBEC, ADAR targets double-stranded RNA; hence, it is active only on plus/minus RNA dimers. In line with this mechanism and in sharp contrast with APOBEC, A>G's and the equivalent T>C's show a similar prevalence: the ratio A>G/T>C is 0.81 $\left(\frac{384/8954}{508/9595}\right)$. This supports the notion that the A>G:T>C mutational process is exquisitely selective for double-stranded RNA, where it can similarly target adenines present on both strands.

### Identification of signature-based clusters

We then clustered the 150 samples with at least 6 always minor mutations (on 1133 total samples) by applying k-means on the normalized low-rank latent NMF matrix and employing standard heuristics to determine the optimal number of clusters (see Methods). As a result, 3 signature-based clusters (SC#1, SC#2, and SC#3) are retrieved, including 61, 49, and 40 samples, respectively (see Figure 3B).

Remarkably, clusters SC#1 and SC#3 are characterized by distinctive signatures, S#1 (dominated by substitution C>T:G>A) and SC#3 (T>C:A>G and T>A:A>T), respectively, whereas cluster SC#2 is characterized by a mixtures of all three signatures. In particular, the samples of the distinct clusters display dissimilar categorical VF distributions (see Figure 3D), pointing at the existence of different host-related mutational processes.

We here recall that samples with a number of always minor variants between 1 and 5 (742 samples, 65.5%) cannot be reliably associated to signature-based clusters, due to the low number of SNVs. For this reason, such samples were considered separately in the analysis and were labeled as cluster SC#4 from now on (Figure 3C).

Importantly, by computing the categorical VF distribution of all minor SNVs with respect to all 96 trinucleotide contexts (i.e., by considering flanking bases), one can notice that clusters SC#1 and SC#2 display profiles that resemble that of the theoretical substitution distribution of the reference genome, thus suggesting that, in such cases, the host-related mutational processes are likely independent from flanking bases. Conversely, SC#3 displays a distribution of T substitutions with prevalent peaks in certain contexts and, especially, in G[T>A]G, A[T>C]G, and C[T>G]T.

We finally note that, due to the possible transmission of minor variants among hosts during infections (see above), signature-based clusters might include both samples with host-related mutational processes and samples with minor variants inherited from infecting hosts.

### Characterization of signature-based clusters

**Figure 4:**
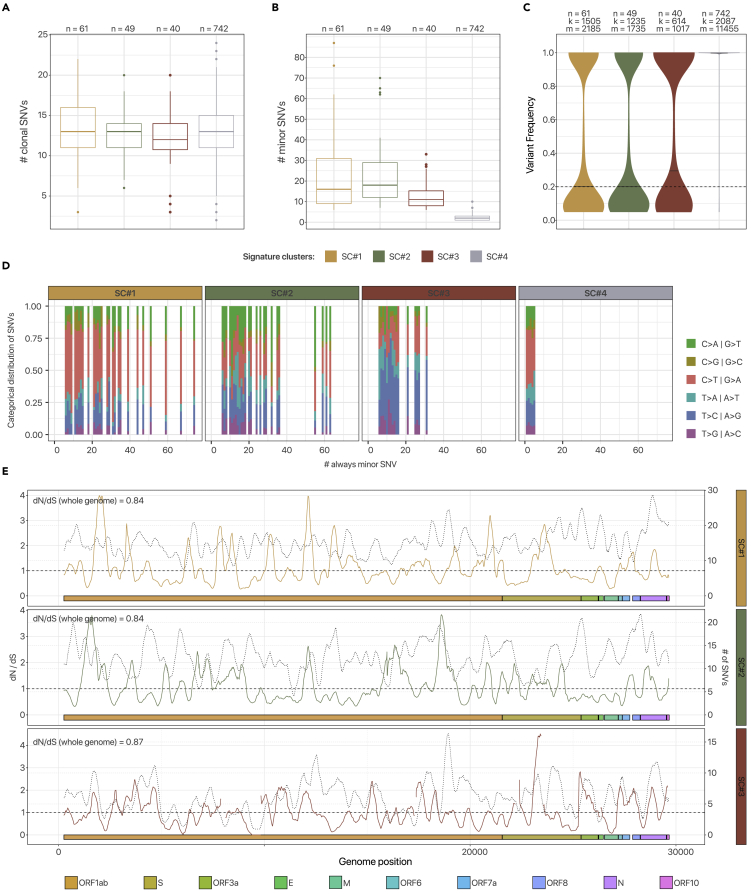
Characterization of signature-based clusters of SARS-CoV-2 samples (A) Distribution of the number of clonal variants with respect to the 4 signature-based clusters described in the text. (B) Distribution of the number of minor variants for the 4 signature-based clusters. (C) Violin plots returning the VF distribution with respect to signature-based clusters (*n* returns the number of samples, *k* the number of distinct SNVs, *m* the number of non-zero entries of the VF matrix). (D) (Average) proportion of substitution classes of always minor variants for all the samples included in the 4 signature-based clusters, grouped and sorted by the number of minor SNVs (e.g., at position 10 of the x axis one can find the average proportion of substitution classes for all samples with 10 minor SNVs). (E) Corrected-for-signatures dN/dS ratio plot, as computed by normalizing the ratio on cluster substitution distribution, on a 300-base sliding window, with respect to signature-based clusters (see Methods). The superimposed dotted line returns the mutational density in each window (rightmost y axis).

We analyzed in depth the intra-host genomic diversity of the samples of the 4 different signature-based clusters. As a first noteworthy result, while the distributions of the number of clonal variants are significantly alike across clusters (Kolmogorov-Smirnov, KS test p>0.20 for 6/6 pairwise comparisons; see Figure 4A and Data S2), clusters SC#1 and SC#2 display a similar distribution of minor variants (KS test p=0.86) but significantly different distributions from the remaining clusters (KS p<0.05 for all remaining pairwise comparisons; see Figure 4B and Data S2). The relative proportion of substitution types for the samples of each signature-based cluster can be found in Figure 4D.

In particular, clusters SC#1 and SC#2 are characterized by a significantly higher number of minor variants (median 16 and 18, mean 22.3 and 22.6, max 87 and 70, for SC#1 and SC#2, respectively). Accordingly, both clusters include a certain proportion of highly mutated samples (with ≥10 minor variants), 44 on 61 and 41 on 49 for SC#1 and SC#2, respectively. This result supports the existence of highly active mutational processes and is consistent with the hypothesis of processes related to APOBEC and ROS. Conversely, cluster SC displays a much lower number of minor variants (median 11, mean 13.2, max 33; 23 samples on 40 with ≥10 variants). This finding hints at the existence of milder spontaneous mutational processes related to ADAR.

Interestingly, the VF distribution for all SNVs highlights a remarkable similarity among signature clusters SC#1, SC#2, and SC#3, with the large majority of variants found either at a high or a low frequency, whereas, by construction, SC#4 is dominated by clonal variants (Figure 4C). Moreover, only minor differences are observed in the distribution of substitutions with respect to SARS-CoV-2 Open Reading Frames (ORFs) (see Figure S3).

Overall, these results reinforce the hypothesis of distinct mutational processes active in different hosts. When clinical data would be available in combination to sequencing data, this will allow us to assess the correlation with clinical outcomes.

### Evidence of purifying selection against signature-related mutagenic processes

To investigate the evolutionary dynamics of SARS-CoV-2, we implemented a corrected-for-signatures version of the dN/dS ratio analysis, i.e., obtained by normalizing the NS/S rate with respect to the theoretical distribution of substitutions detected in each cluster, as suggested in a different context in Van den Eynden and Larsson (2017).

Interestingly, the corrected dN/dS ratio computed on the genome coding regions (i.e., =29133 basis) is equal to 0.84, 0.84, and 0.87 for the three signature-based clusters, respectively, and suggests the existence of purifying selection for all signature-related mutational processes.

We refined the analysis via a 300-base sliding window approach. On the one hand, the analysis of the mutational density confirms that the large majority of variants is indeed observed in purified regions of the genome. On the other hand, however, the variation of the corrected dN/dS ratio across the genome shows that some regions exhibit a ratio significantly larger than 1. This phenomenon, which is particularly evident in signature-based clusters SC#1 and SC#2, hints at possible positive selection processes affecting specific genomic regions and deserves further investigations.

### Phylogenomic model of SARS-CoV-2 reveals transmission of minor variants and homoplasies

**Figure 5:**
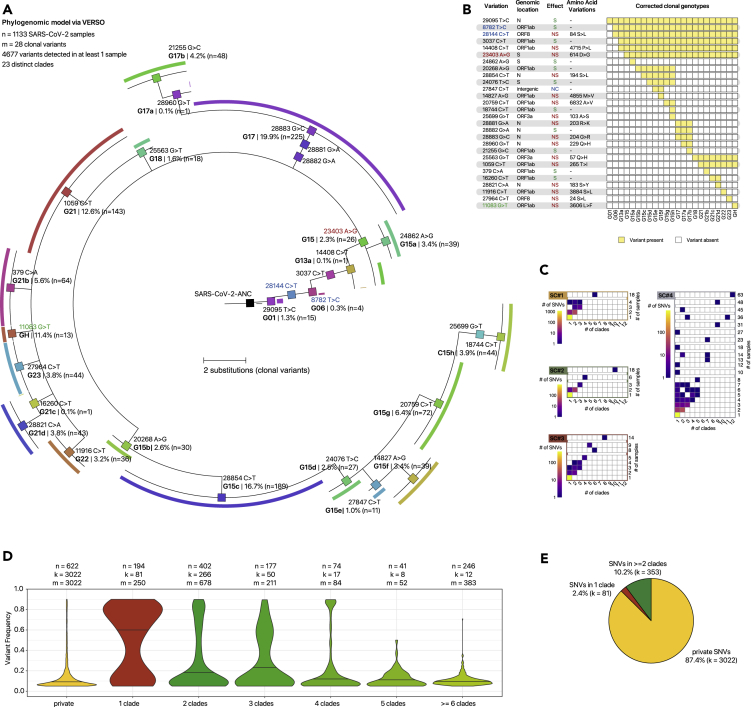
Phylogenomic model of 1133SARS-CoV-2 samples of via VERSO – data set #1 (NCBI BioProject: PRJNA645906) (A)The phylogenetic tree returned by VERSO (Ramazzotti et al., 2021) considering 28 clonal variants (VF >0.90) detected in at least 3% of the 1133 samples of the data set is displayed. Colors mark the 23 distinct clades identified by VERSO, which are associated to corrected clonal genotypes. Genotype labels are consistent with (Ramazzotti et al., 2021), whereas in Supplementary File S3, one can find the mapping with the lineage nomenclature proposed in Rambaut et al. (2020). Samples with identical corrected clonal genotypes are grouped in polytomies (visualization via FigTree (Rambaut, 2009)). The black colored sample represents the SARS-CoV-2-ANC reference genome. (B) Heatmap returning the composition of the 23 corrected clonal genotypes returned by VERSO. Clonal SNVs are annotated with mapping on ORFs, synonymous (S), nonsynonymous (NS) and non-coding (NC) states, and related amino acid substitutions. Variants g.8782T>C (*ORF1ab*, synonymous) and g.28144C>T (*ORF8*, p.84S>L) are colored in blue, variant g.23403 A>G (*S*, p.614 D>G) in red, homoplastic variant g.11083G>T (*ORF1ab*, p.3606L>F) in green. (C) Heatmaps displaying the count of minor variants with respect to the number of clades and samples in which they are found, grouped by signature-based cluster (e.g., at row 3 and column 5, the color represents the number of SNVs found in 3 clades and 5 samples). (D) Violin plots returning the VF distribution of all minor variants, with respect to the number of clades in which they are found (the first violin plot is associated to variants privately detected in single samples). *n* returns the number of samples, *k* the number of distinct SNVs, *m* the number of non-zero entries of the VF matrix. (E) Pie chart returning the proportion of minor variants privately detected in single samples, detected in multiple samples of the same clade, and in multiple samples of independent clades.

We employed VERSO (Ramazzotti et al., 2021) to reconstruct a robust phylogeny of samples from the binarized VF profiles of the 28 clonal variants (VF >90%) detected in at least 3% of the data set. In Figure 5A, one can see the output phylogenetic tree, which describes the existence of 23 clades and in which samples with identical corrected clonal genotype are grouped in polytomies (see Figure 5B and the Methods section for further details). The mapping between clonal genotype labels and the lineage dynamic nomenclature proposed in Rambaut et al. (2020) and generated via pangolin 2.0 (O'Toole et al., 2020) is included in Data S3, whereas the phylogenetic model returned via MrBayes (Ronquist et al., 2012) on data set #1 is displayed in Figure S5 (see Methods).

Interestingly, SNV g.29095T>C (mapped on ORF *N*, synonymous) appears to be the earliest evolutionary event from reference genome SARS-CoV-2-ANC (Ramazzotti et al., 2021). All downstream clades belong to type B type (Forster et al., 2020; Tang et al., 2020), as determined by presence of mutations g.8782T>C (*ORF1ab*, synonymous) and g.28144C>T (*ORF1ab*, p.84S>L). Importantly, we note that variant g.23403A>G (*S*, p.614D>G), whose correlation with viral transmissibility was investigated in depth (Lokman et al., 2020; Daniloski et al., 2020; Korber et al., 2020; Zhou et al., 2020a; Grubaugh et al., 2020; Plante et al., 2020), is found in 18 clades, which include 1113 samples of the data set.

In addition, the model unveils the presence of a number of homoplasies, as a few clonal variants are observed in independent clades and, especially, mutation g.11083G>T (*ORF1ab*, p.3606L>F), which was investigated in a number of recent studies on SARS-CoV-2 evolution (van Dorp et al., 2020; Ramazzotti et al., 2021), and is observed in 42 samples and 8 distinct clades. One might hypothesize that such SNVs have spontaneously emerged in unrelated samples and were selected either due to some functional advantage or alternatively to the combination of founder and stochastic effects involved in variant transmission during infections, which might lead certain minor SNVs transiting to clonality in the population (see above).

As extensively discussed in Ramazzotti et al., 2021, while all the clonal variants of a host are most likely transmitted during an infection, the extent of transmission of minor variants is still baffling and is highly influenced by bottlenecks, founder effects, and stochasticity (Gutierrez et al., 2012; Domingo et al., 2012). Simultaneous infections of the same host from multiple individuals harboring distinct viral lineages (also named superinfections) might in principle affect variant clonality, yet their occurrence is extremely rare (Lythgoe et al., 2020).

For this reason, we quantified the number of minor variants (VF ≤90%)

1. privately detected in single samples and which are most likely spontaneously emerged via host-related mutational processes;
2. found in multiple samples of the same clade, which might be either (a) spontaneously emerged or (b) transferred from other hosts via infection chains; and
3. observed in multiple samples of independent clades (i.e., homoplasies) and which might be due to (a) positive selection of the variants due to some functional advantage, in a scenario of parallel/convergent evolution, (b) mutational hotspots, i.e., SVNs falling in mutation-prone sites or regions of the viral genome, (c) phantom mutations due to sequencing artifacts (Bandelt et al., 2002), and (d) complex transmission dynamics involving founder effects and stochasticity, which may allow certain minor variants to transit to clonality, eventually leading to a clonal genotype transmutation (see above).

In our case, we observe that 87.4% of minor variants are observed as private of single samples, 2.4% in multiple samples of the same clade, and 10.2% are detected in samples belonging to distinct clades (Figure 5E). Importantly, significantly different VF distributions are observed, and, especially, an approximately monotonic decrease of the median VF is detected with respect to the number of clades in which minor variants are found (Figure 5D). Important conclusions can be drawn from these results.

Apparently, the large majority of minor SVNs spontaneously emerges in single samples, likely due to signature-based mutational processes. Yet, the VF distribution of private minor SNVs suggests that, as expected, most of such variants are indeed purified in the population.

Accordingly, the hypothesis of transmission of minor variants during infections is supported by the significantly larger VF of (the fewer) minor variants found in multiple samples of the same clade, as this effect is most likely due to transmission bottleneck effects (Gutierrez et al., 2012; Domingo et al., 2012).

In addition, the progressively smaller VF of minor variants observed in samples of independent clades and which are likely more distant in the infection chain hints at the noteworthy presence of mutational hotspots and of phantom mutations related to sequencing artifacts (Bandelt et al., 2002). In all scenarios, the presence of positively selected variant cannot be excluded but requires ad hoc investigations.

Interestingly, one can refine the analysis by focusing on distinct signature-based clusters, for instance, by pinpointing variants likely related to mutational hotspots or phantom mutations: see, e.g., variant g.8651A>C (*ORF1ab*, p.2796M>L) which is observed in 63 samples and 12 clades (Figure 5C).

### Validation – data sets #2−5

We employed 4 independent data sets (NCBI BioProjects: PRJNA625551, PRJNA633948, PRJNA636748, and PRJNA647529; see Methods for details) to validate the presence of the discovered mutational signatures. Specifically, we performed signature assignment with respect to the discovered signatures on 141, 23, 17, and 14 high-quality samples showing ≥6 always minor variants in each data set, respectively.

**Figure 6:**
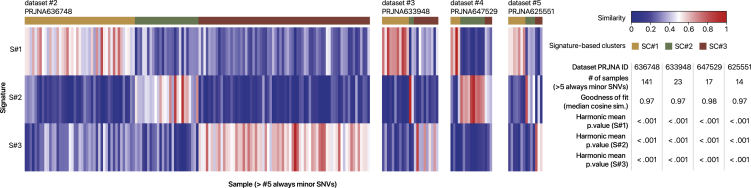
Validation on data sets #2−5 (NCBI BioProject: PRJNA636748, PRJNA633948, PRJNA647529, and PRJNA625551) Heatmap returning the clustering of 141, 23, 17, and 14 samples of data sets #2−5 with ≥6 always minor variants (of the data set), computed via k-means on the low-rank latent NMF matrix on the three signatures discovered on data set ≈13% (see Methods). The goodness of fit in terms of median cosine similarity between observations and predictions and the harmonic mean p value of the one-sided Mann-Whitney U test on bootstrap re-sampling are shown for all signatures (see Methods).

Three signature-based clusters are found for all data sets and explain more that 97% of the variance in all cases, with highly significant p values (see Figure 6). Such clusters are related to combinations of signatures consistently to the analysis presented in the text and display alike distributions of minor SVNs (see Figure S6).

These important results prove the generality of our findings and strongly support the hypothesis of distinct mutational processes active in distinct groups of samples.

## Discussion

Standard (phylo)genomic analyses of viral consensus sequences might miss useful information to investigate the elusive mechanisms of viral evolution within hosts and of transmission among hosts. In this respect, raw sequencing data of viral samples can be effectively employed to deliver a high-resolution picture of intra-host heterogeneity, which might underlie different clinical outcomes and affect the efficacy of anti-viral therapies. This aspect is vital especially during the critical phases of an outbreak, as experimental hypotheses are urgently needed to deliver effective prognostic, diagnostic, and therapeutic strategies for infected patients.

We here presented one of the largest up-to-date quantitative analyses of intra-host genomic diversity of SARS-CoV-2, which revealed that the large majority of samples present a complex genomic composition, likely due to the interplay between host-related mutational processes and transmission dynamics.

In particular, we here proved the existence of mutually exclusive viral mutational signatures, i.e., nucleotide substitution patterns, which show that different hosts respond to SARS-CoV-2 infections in different ways, likely ruled by APOBEC, ROS, or ADAR-related processes.

The corrected-for-signatures dN/dS analysis shows that such numerous low-frequency variants tend to be purified in the population whereas, conversely, a certain number of variants appear to consolidate. In particular, due to the still obscure combination of bottleneck effects and selection phenomena, certain variants appear to transit to clonality in the population, eventually leading to the definition of new clonal genotypes. Once become clonal, mutations tend to accumulate in the population, as proven by a statistically significant increase of genomic diversity, and might be used to reconstruct robust models of viral evolution via standard phylogenetic approaches.

The analysis of homoplasies, i.e., minor variants shared across distinct clades and unlikely due to infection events, demonstrates that a high number of mutations can independently emerge in multiple samples, due to mutational hotspots often related to signatures or, possibly, to positive (functional) selection.

In addition, the relatively higher VF of minor variants shared by multiple samples of the same clades supports the hypothesis of transmission during infections.

To conclude, we advocate the release of a larger number of raw sequencing data sets, especially in combination with clinical data, in order to investigate the relation among the discovered host-specific processes and clinical outcomes.

## Limitations of the study

### Reference genome

Different reference genomes have been employed for variant calling in the investigation of the origin and evolution of SARS-CoV-2. For instance, sequence EPI_ISL_405839 was used, e.g., in Bastola et al. (2020) and sequence EPI_ISL_402125, e.g., in Andersen et al. (2020). As detailed in the Methods section, here we employed as reference the sequence SARS-CoV-2-ANC, which was identified in Ramazzotti et al., 2021 as a likely ancestral SARS-CoV-2 genome. Clearly, the use of different, albeit mostly overlapping, reference genomes can influence downstream analyses and, especially, the inference of the first evolutionary steps of the phylogenomic model, which should be therefore considered with caution. However, in our specific case, the employment of any of such reference genomes does not impact the identification and characterization of mutational signatures since the SNVs that distinguish such sequences are found as clonal in at least one sample of the data set and, accordingly, are excluded from the analysis.

### Quasispecies composition

As discussed in the Introduction section, the analysis of raw sequencing data might be used to characterize the quasispecies architecture of single samples. To this end, a plethora of sophisticated computational methods for the characterization of the quasispecies composition of single samples is available, e.g., (Prosperi and Salemi, 2012; Giallonardo et al., 2014; Töpfer et al., 2014; Barik et al., 2018), and was recently reviewed in Knyazev et al. (2020). In the phylogenomic analysis included in this work, we decided to restrict the analysis on clonal variants that, by definition, are present in most of (or all) the quasispecies of a given sample. This allows us to provide a coarse-grained picture of the main steps of SARS-CoV-2 evolution and, at the same time, to investigate the possible transmission of minor variants, which are related to scarcely prevalent and rare quasispecies. It would be worth investigating how the combination of more sophisticated methods for quasispecies deconvolution and of our approach for mutational signatures analysis may improve the overall comprehension of SARS-CoV-2 diversity, adaptability, and evolution.

### Data set quality

It was recently noted that some currently available SARS-CoV-2 data sets might present quality issues, especially with respect to low-frequency variants (De Maio et al., 2020). For this reason, the results of any computational pipeline should be, in principle, validated on data sets for which the ground truth is known. In our case and given the current shortage of SARS-CoV-2 benchmark data sets, we decided to validate the discovery and characterization of the mutational signatures on 4 different data sets, generated from independent laboratories worldwide, so to ensure the generality of the results obtained via our framework (see the Validation section).

### Indels

The evolution of SARS-CoV-2 is characterized by the presence of a significant number of insertions and deletions (Koyama et al., 2020), which are being cataloged via COV-Glue (Singer et al., 2020). In this work, we focused on the analysis of SNVs, as this allows us to discover and characterize statistically significant host-related mutational signatures. Despite being beyond of the scope of the current work, it might be worth investigating the origination, evolution, and transmission of indels as well.

## Resource availability

### Lead contact

Alex Graudenzi, Institute of Molecular Bioimaging and Physiology, Consiglio Nazionale delle Ricerche (IBFM-CNR), via F.lli Cervi, 93, 20,090 Segrate, Milan, Italy. alex.graudenzi@ibfm.cnr.it.

### Material availability

This study did not generate new unique reagents.

### Data and code availability

The source code used to replicate all the analyses is available at this link: https://github.com/BIMIB-DISCo/SARS-CoV-2-IHMV. VERSO can be downloaded at this link: https://github.com/BIMIB-DISCo/VERSO. Additional supplemental items are available from Mendeley Data at: https://doi.org/10.17632/vwc9jx5jfm.2.

## Methods

All methods can be found in the accompanying Transparent methods supplemental file.

## Supplemental information

Document S1. Transparent methods and Figures S1–S6

Data S1. File including the VF profiles of all samples of Dataset #1, Related to Figure 1

Data S2. Result of the Kolmogorov-Smirnov (KS) test on clonal variant distributions among all signature-based clusters, Related to Figure 4

Data S3. Mapping between the clonal genotype labels of the phylogenomic models returned via VERSO STEP #1 on Dataset #1 and the lineage dynamic nomenclature proposed by Rambaut et al. (2020), generated via pangolin 2.0 (O’Toole et al., 2020), Related to Figure 5

## References

[bib1] Alexandrov L.B., Nik-Zainal S., Wedge D.C., Aparicio S.A., Behjati S., Biankin A.V., Bignell G.R., Bolli N., Borg A., Børresen-Dale A.L. (2013). Signatures of mutational processes in human cancer. Nature.

[bib2] Alexandrov L.B., Kim J., Haradhvala N.J., Huang M.N., Ng A.W.T., Wu Y., Boot A., Covington K.R., Gordenin D.A., Bergstrom E.N. (2020). The repertoire of mutational signatures in human cancer. Nature.

[bib3] Andersen K.G., Rambaut A., Lipkin W.I., Holmes E.C., Garry R.F. (2020). The proximal origin of SARS-CoV-2. Nat. Med..

[bib4] Bandelt H.J., Quintana-Murci L., Salas A., Macaulay V. (2002). The fingerprint of phantom mutations in mitochondrial DNA data. Am. J. Hum. Genet..

[bib5] Barik S., Das S., Vikalo H. (2018). QSdpR: viral quasispecies reconstruction via correlation clustering. Genomics.

[bib6] Bastola A., Sah R., Rodriguez-Morales A.J., Lal B.K., Jha R., Ojha H.C., Shrestha B., Chu D.K., Poon L.L., Costello A. (2020). The first 2019 novel coronavirus case in Nepal. Lancet Infect. Dis..

[bib7] Brunet J.P., Tamayo P., Golub T.R., Mesirov J.P. (2004). Metagenes and molecular pattern discovery using matrix factorization. Proc. Natl. Acad. Sci. U S A.

[bib8] Capobianchi M.R., Rueca M., Messina F., Giombini E., Carletti F., Colavita F., Castilletti C., Lalle E., Bordi L., Vairo F. (2020). Molecular characterization of SARS-CoV-2 from the first case of COVID-19 in Italy. Clin. Microbiol. Infect..

[bib9] Chan J.M., Carlsson G., Rabadan R. (2013). Topology of viral evolution. Proc. Natl. Acad. Sci. U S A.

[bib10] Daniloski Z., Guo X., Sanjana N.E. (2020). The D614G mutation in SARS-CoV-2 spike increases transduction of multiple human cell types. bioRxiv.

[bib11] David S.S., O’Shea V.L., Kundu S. (2007). Base-excision repair of oxidative DNA damage. Nature.

[bib12] Deng X., Gu W., Federman S., du Plessis L., Pybus O.G., Faria N., Wang C., Yu G., Bushnell B., Pan C.Y. (2020). Genomic surveillance reveals multiple introductions of SARS-CoV-2 into northern California. Science.

[bib13] Domingo E., Martínez-Salas E., Sobrino F., de la Torre J.C., Portela A., Ortín J., López-Galindez C., Pérez-Breña P., Villanueva N., Nájera R. (1985). The quasispecies (extremely heterogeneous) nature of viral RNA genome populations: biological relevance-a review. Gene.

[bib14] Domingo E., Sheldon J., Perales C. (2012). Viral quasispecies evolution. Microbiol. Mol. Biol. Rev..

[bib15] van Dorp L., Acman M., Richard D., Shaw L.P., Ford C.E., Ormond L., Owen C.J., Pang J., Tan C.C., Boshier F.A. (2020). Emergence of genomic diversity and recurrent mutations in SARS-CoV-2. Infect. Genet. Evol..

[bib16] Van den Eynden J., Larsson E. (2017). Mutational signatures are critical for proper estimation of purifying selection pressures in cancer somatic mutation data when using the dn/ds metric. Front. Genet..

[bib17] Forster P., Forster L., Renfrew C., Forster M. (2020). Phylogenetic network analysis of SARS-CoV-2 genomes. Proc. Natl. Acad. Sci. U S A.

[bib18] Giallonardo F.D., Töpfer A., Rey M., Prabhakaran S., Duport Y., Leemann C., Schmutz S., Campbell N.K., Joos B., Lecca M.R. (2014). Full-length haplotype reconstruction to infer the structure of heterogeneous virus populations. Nucleic Acids Res..

[bib19] Di Giorgio S., Martignano F., Torcia M.G., Mattiuz G., Conticello S.G. (2020). Evidence for host-dependent RNA editing in the transcriptome of SARS-CoV-2. Sci. Adv..

[bib20] Grubaugh N.D., Hanage W.P., Rasmussen A.L. (2020). Making sense of mutation: what D614G means for the COVID-19 pandemic remains unclear. Cell.

[bib21] Gutierrez S., Yvon M., Pirolles E., Garzo E., Fereres A., Michalakis Y., Blanc S. (2012). Circulating virus load determines the size of bottlenecks in viral populations progressing within a host. PLoS Pathog..

[bib22] Knyazev S., Hughes L., Skums P., Zelikovsky A. (2020). Epidemiological data analysis of viral quasispecies in the next-generation sequencing era. Brief. Bioinform..

[bib23] Korber B., Fischer W., Gnanakaran S., Yoon H., Theiler J., Abfalterer W., Hengartner N., Giorgi E., Bhattacharya T., Foley B. (2020). Tracking changes in SARS-CoV-2 spike: evidence that D614G increases infectivity of the COVID-19 virus. Cell.

[bib24] Koyama T., Platt D., Parida L. (2020). Variant analysis of SARS-CoV-2 genomes. Bull. World Health Organ..

[bib25] Li X., Wang W., Zhao X., Zai J., Zhao Q., Li Y., Chaillon A. (2020). Transmission dynamics and evolutionary history of 2019-nCoV. J. Med. Virol..

[bib26] Lokman S.M., Rasheduzzaman M., Salauddin A., Barua R., Tanzina A.Y., Rumi M.H., Hossain M.I., Siddiki A.Z., Mannan A., Hasan M.M. (2020). Exploring the genomic and proteomic variations of SARS-CoV-2 spike glycoprotein: a computational biology approach. Infect. Genet. Evol..

[bib27] Lu J., du Plessis L., Liu Z., Hill V., Kang M., Lin H., Sun J., François S., Kraemer M.U., Faria N.R. (2020). Genomic epidemiology of SARS-CoV-2 in guangdong province, China. Cell.

[bib28] Lucas M., Karrer U., Lucas A., Klenerman P. (2001). Viral escape mechanisms–escapology taught by viruses. Int. J. Exp. Pathol..

[bib29] Lythgoe K.A., Hall M.D., Ferretti L., de Cesare M., MacIntyre-Cockett G., Trebes A., Andersson M., Otecko N., Wise E.L., Moore N. (2020). Shared SARS-CoV-2 diversity suggests localised transmission of minority variants. bioRxiv.

[bib30] De Maio N., Walker C., Borge R., Weilguny L., Slodkowick G., Goldmand N. (2020). Issues with SARS-CoV-2 Sequencing Data. https://virological.org/.

[bib31] Molteni C., Principi N., Esposito S. (2014). Reactive oxygen and nitrogen species during viral infections. Free Radic. Res..

[bib32] Ni M., Chen C., Qian J., Xiao H.X., Shi W.F., Luo Y., Wang H.Y., Li Z., Wu J., Xu P.S. (2016). Intra-host dynamics of ebola virus during 2014. Nat. Microbiol..

[bib33] Nishikura K. (2010). Functions and regulation of RNA editing by ADAR deaminases. Annu. Rev. Biochem..

[bib34] Novella I.S., Domingo E., Holland J.J. (1995). Rapid viral quasispecies evolution: implications for vaccine and drug strategies. Mol. Med. Today.

[bib35] O’Toole A., McCrone J., Scher E. (2020). Pangolin 2.0. https://github.com/cov-lineages/pangolin.

[bib36] Park D., Huh H.J., Kim Y.J., Son D.S., Jeon H.J., Im E.H., Kim J.W., Lee N.Y., Kang E.S., Kang C.I. (2016). Analysis of intrapatient heterogeneity uncovers the microevolution of middle east respiratory syndrome coronavirus. Mol. Case Stud..

[bib37] Plante J.A., Liu Y., Liu J., Xia H., Johnson B.A., Lokugamage K.G., Zhang X., Muruato A.E., Zou J., Fontes-Garfias C.R. (2020). Spike mutation D614G alters SARS-CoV-2 fitness. Nature.

[bib38] Poon L.L., Song T., Rosenfeld R., Lin X., Rogers M.B., Zhou B., Sebra R., Halpin R.A., Guan Y., Twaddle A. (2016). Quantifying influenza virus diversity and transmission in humans. Nat. Genet..

[bib39] Popa A., Genger J.W., Nicholson M.D., Penz T., Schmid D., Aberle S.W., Agerer B., Lercher A., Endler L., Colaço H. (2020). Genomic epidemiology of superspreading events in Austria reveals mutational dynamics and transmission properties of SARS-CoV-2. Sci. Transl. Med..

[bib40] Prosperi M.C., Salemi M. (2012). QuRe: software for viral quasispecies reconstruction from next-generation sequencing data. Bioinformatics.

[bib41] Ramazzotti D., Angaroni F., Maspero D., Gambacorti-Passerini C., Antoniotti M., Graudenzi A., Piazza R. (2021). VERSO: a comprehensive framework for the inference of robust phylogenies and the quantification of intra-host genomic diversity of viral samples. Patterns.

[bib42] Rambaut A. (2009). Figtree v1. 3.1. http://tree.bio.ed.ac.uk/software/figtree/.

[bib43] Rambaut A., Holmes E.C., O’Toole Á., Hill V., McCrone J.T., Ruis C., du Plessis L., Pybus O.G. (2020). A dynamic nomenclature proposal for SARS-CoV-2 lineages to assist genomic epidemiology. Nat. Microbiol..

[bib44] Reshi M.L., Su Y.C., Hong J.R. (2014). RNA viruses: ROS-mediated cell death. Int. J. Cell Biol..

[bib45] Ronquist F., Teslenko M., van der Mark P., Ayres D.L., Darling A., Höhna S., Larget B., Liu L., Suchard M.A., Huelsenbeck J.P. (2012). MrBayes 3.2: efficient Bayesian phylogenetic inference and model choice across a large model space. Syst. Biol..

[bib46] Rose R., Nolan D.J., Moot S., Feehan A., Cross S., Garcia-Diaz J., Lamers S.L. (2020). Intra-host site-specific polymorphisms of SARS-CoV-2 is consistent across multiple samples and methodologies. medRxiv.

[bib47] Seemann T., Lane C.R., Sherry N.L., Duchene S., Gonçalves da Silva A., Caly L., Sait M., Ballard S.A., Horan K., Schultz M.B. (2020). Tracking the covid-19 pandemic in Australia using genomics. Nat. Commun..

[bib48] Sharma S., Patnaik S.K., Taggart R.T., Kannisto E.D., Enriquez S.M., Gollnick P., Baysal B.E. (2015). Apobec3a cytidine deaminase induces rna editing in monocytes and macrophages. Nat. Commun..

[bib49] Shen Z., Xiao Y., Kang L., Ma W., Shi L., Zhang L., Zhou Z., Yang J., Zhong J., Yang D. (2020). Genomic diversity of SARS-CoV-2 in coronavirus disease 2019 patients. Clin. Infect. Dis..

[bib50] Shu Y., McCauley J. (2017). GISAID: global initiative on sharing all influenza data–from vision to reality. Eurosurveillance.

[bib51] Simmonds P. (2020). Rampant C -> U hypermutation in the genomes of SARS-CoV-2 and other coronaviruses: causes and consequences for their short-and long-term evolutionary trajectories. MSphere.

[bib52] Singer J., Gifford R., Cotten M., Robertson D. (2020). CoV-GLUE: a web application for tracking SARS-CoV-2 genomic variation. Preprints.

[bib53] Tang X., Wu C., Li X., Song Y., Yao X., Wu X., Duan Y., Zhang H., Wang Y., Qian Z. (2020). On the origin and continuing evolution of SARS-CoV-2. Natl. Sci. Rev..

[bib54] Töpfer A., Marschall T., Bull R.A., Luciani F., Schönhuth A., Beerenwinkel N. (2014). Viral quasispecies assembly via maximal clique enumeration. PLoS Comput. Biol..

[bib55] Wölfel R., Corman V.M., Guggemos W., Seilmaier M., Zange S., Müller M.A., Niemeyer D., Jones T.C., Vollmar P., Rothe C. (2020). Virological assessment of hospitalized patients with COVID-2019. Nature.

[bib56] Woo P.C., Wong B.H., Huang Y., Lau S.K., Yuen K.Y. (2007). Cytosine deamination and selection of cpg suppressed clones are the two major independent biological forces that shape codon usage bias in coronaviruses. Virology.

[bib57] World Health Organization (WHO) (2020). Coronavirus Disease 2019 (COVID-19): Situation Report. https://www.who.int/emergencies/diseases/novel-coronavirus-2019/situation-reports.

[bib58] Wu F., Zhao S., Yu B., Chen Y.M., Wang W., Song Z.G., Hu Y., Tao Z.W., Tian J.H., Pei Y.Y. (2020). A new coronavirus associated with human respiratory disease in China. Nature.

[bib59] Xiao K., Zhai J., Feng Y., Zhou N., Zhang X., Zou J.J., Li N., Guo Y., Li X., Shen X. (2020). Isolation of SARS-CoV-2-related coronavirus from malayan pangolins. Nature.

[bib60] Xu D., Zhang Z., Wang F.S. (2004). Sars-associated coronavirus quasispecies in individual patients. N. Engl. J. Med..

[bib61] Zhou B., Thao T.T.N., Hoffmann D., Taddeo A., Ebert N., Labroussaa F., Pohlmann A., King J., Portmann J., Halwe N.J. (2020). SARS-CoV-2 spike D614G variant confers enhanced replication and transmissibility. bioRxiv.

[bib62] Zhou P., Yang X.L., Wang X.G., Hu B., Zhang L., Zhang W., Si H.R., Zhu Y., Li B., Huang C.L. (2020). A pneumonia outbreak associated with a new coronavirus of probable bat origin. Nature.

