# Supplementary material for: Mutational signatures and heterogeneous host response revealed via large-scale characterization of SARS-CoV-2 genomic diversity
Source: iScience. 2021 Jan 28;24(2):102116. doi: 10.1016/j.isci.2021.102116 (PMC7842190; doi:10.1016/j.isci.2021.102116)
Supplement: Document S1. Transparent methods and Figures S1–S6 [file mmc1.pdf]

iScience, Volume *24*

Supplemental Information

Mutational signatures and heterogeneous host response revealed via large-scale characterization of SARS-CoV-2 genomic diversity

Alex Graudenzi, Davide Maspero, Fabrizio Angaroni, Rocco Piazza, and Daniele Ramazzotti

List of Figures

S1 Quality check – Dataset #1 PRJNA645906. Related to Figure 1 5
S2 Distribution of SNVs detected on SARS-CoV-2 genome – Dataset #1 PRJNA645906. Related to Figure 2 6
S3 Distribution of substitution types on SARS-CoV-2 ORFs – Dataset #1 PRJNA645906. Related to Figure 2. 7
S4 Signature analysis: Cophenetic correlation coefficient, Explained variance, goodness of fit – Dataset #1 PRJNA645906. Related to Figure 3. 7
S5 Phylogenomic model returned by MrBayes – Dataset #1 PRJNA645906. Related to Figure 5 8
S6 Validation datasets. Related to Figure 6 9

Transparent Methods

Datasets

Dataset #1. We analyzed a dataset comprising 1188 samples from NCBI BioProject with accession number PRJNA645906. For all samples, Illumina AMPLICON sequencing high-coverage raw data are provided; all patients were located in California, United States. Within this dataset, we considered for our analyses, 1133 high-quality samples having coverage ≥ 20 in at least 75% of the virus genome.

Datasets #2−5 (validation). We considered 4 additional datasets for validation, NCBI BioProject with accession numbers PRJNA625551 (United States, 272 AMPLICON samples), PRJNA633948 (Australia, 203 AMPLICON samples), PRJNA636748 (South Africa, 408 AMPLICON samples), PRJNA647529 (Israel, 212 AMPLICON samples). We applied the same QC filters to these datasets (coverage ≥ 20 in at least 75% of virus genome), to obtain four validation set including a total of 953 samples.

SNVs calling. For all datasets, we downloaded SRA files and converted them to FASTQ files using SRA toolkit. Following Ramazzotti et al. (2020), we used Trimmomatic (version 0.39) to remove positions at low quality from the RNA sequences, using the following settings: LEADING:20 TRAILING:20 SLIDINGWINDOW:4:20 MINLEN:40.

We used bwa mem (version 0.7.17) to map reads to the reference genome SARS-CoV-2-ANC, which was recently released in Ramazzotti et al. (2020). SARS-CoV-2-ANC is identical to EPI_ISL_405839 (Bastola et al., 2020) and EPI_ISL_402125 (Andersen et al., 2020) reference genomes on 29865 (out of 29870) genome locations (99.9%), includes the polyA tail of the latter genome (33 bases), and has haplotype TCTCT at locations 8782, 9561, 15607, 28144 and 29095, as observed in both the Bat-CoV-RaTG13 (sequence EPI_ISL_402131) (Zhou et al., 2020) and Pangolin-CoV (sequence EPI_ISL_410721) (Andersen et al., 2020; Xiao et al., 2020) genomes.

We then generated sorted BAM files from bwa mem results with SAMtools (version 1.6) and removed duplicates with Picard (version 2.22.1). Variant calling was performed generating mpileup files with SAMtools and then using VarScan (min-var-freq parameter set to 0.01) (Koboldt et al., 2012).

We finally verified the absence of any possible bias in the detection of minor variants due to sequencing artifacts. As one can see in Supplementary Fig. S1, no correlation between the number of SNVs and both total coverage and the median coverage is observed ($R^2 < 0.04$ in both cases), which proves the good quality of the calls.

Signatures analysis

The analysis was performed on always minor variants (VF $> 5\%$ and $\leq 90\%$ in all samples in which they are detected), in order to ensure that the considered variants are not due to transmission, but are likely emerged in the host. In such way, we could associate to each discovered signature a mechanism causing variants in the viral genome related to the specific host.

Signatures decomposition was formulated as a Non-negative Matrix Factorization problem (NMF) (Brunet et al., 2004). Given n samples, r possible substitution classes (e.g., C>T:G>A) and s signatures, we can define the following objects:

- the input data matrix **D**, a $n \times r$ dimensional matrix, where every element $d_{i,j}$ represents the number of SNVs with substitution class j in the ith sample. Note that $d_{i,j} \in \mathbb{R}^+$;
- the low-rank latent NMF matrix **A**, a $n \times s$ dimensional matrix , where every element $a_{i,j}$ represents the linear combination coefficient of signature j in sample i (also *exposure* of the ith sample to signature j (Alexandrov et al., 2013)). Note that $a_{i,j} \in \mathbb{R}^+$;
- the signature (or basis) matrix **B**, a $s \times r$ dimensional matrix, where every row is a categorical distribution of all substitution classes in each signature. For this matrix we assume that every row must sum up to 1, then $b_{i,j} \in \{0, 1\}$ and $\sum_{i=1}^{r} b_{i,j} = 1$.

In particular, we here considered 6 substitution classes ($r = 6$) by merging equivalent substitution types, namely G>T:C>A, G>C:C>G, G>A:C>T, A>T:T>A, A>G:T>C and A>C:T>G.

Problem 1 *(Signature decomposition): Given the data matrix* **D**, *we aim at finding the NMF latent matrix* **A** *and the signature matrix* **B**, *such that* $||\mathbf{D} - \mathbf{A} \cdot \mathbf{B}||_2$ *is minimum.*

To solve the stated problem, we here performed a total of 100 independent NMF runs with standard update (Brunet et al., 2004), for solutions at ranks varying from 1 to 6, where initial solutions were randomly initialized; for each run a total of 20 iterations were performed where signatures and their assignments to samples were iteratively estimated by non-negative least squares (Chen and Plemmons, 2010). The final solution was constructed as the consensus of the 100 runs (Brunet et al., 2004).

We then employed multiple state-of-the-art approaches to assess the optimal rank (optimal number of signatures s) for the NMF decomposition. We first assess the stability of NMF results over the 100 runs, with the idea that stable solutions are preferable to unstable ones; to this extent, we computed the average Cophenetic correlation coefficient (Brunet et al., 2004), which showed high stability (0.998) at rank equal to 3 (see Supplementary Fig. S3A). Furthermore, we also evaluated the goodness of fit of NMF solutions at different ranks, with rank equals to 3 being able to explain 96.54% of variance in the data (see Supplementary Fig. S3B); finally, we report as Supplementary Fig. S3C the average cosine similarity between observations and predictions by NMF with rank equals to 3, showing a plateau with correlation equals 0.973 (Lal et al., 2020). All of this supports 3 as the optimal rank for our decomposition problem and the presence of 3 distinct mutational signatures in our data.

Identification of signature-based clusters. In order to identify clusters of samples possibly affected in different proportions by the discovered mutational signatures, we considered the low-rank latent NMF matrix **A** defined above.

Specifically, we first normalized **A** such that each row of the matrix sums up to 1 and then computed the euclidean distance among each pair of samples. We next performed Principal Component Analysis (PCA) on the distance matrix to estimate the optimal number of clusters present in our data. In

detail, the analysis of the eigenvalues of the distance matrix shows that 3 components explain $> 99\%$ of the variance, followed by a plateau. Accordingly, we performed k-means clustering with $k = 3$ on the normalized **A** matrix to discover the signature-based clusters.

Assessment of signatures significance. We assessed the statistical significance of samples exposure to signatures by bootstrap. Namely, for each sample we performed 1000 bootstrap re-sampling from their observed variants cumulative distributions and assigned signatures to each bootstrap dataset in order to obtain 1000 independent assignments for each sample. Then, for each signature we computed a p-value by Mann-Whitney U test to verify the hypothesis that such signature was contributing to more than 5% variants (one-sided test). A p-value assessing the significance of each signature was computed as the harmonic mean of the Mann-Whitney U test p-values (Wilson, 2019).

Corrected-for-signatures dN/dS analysis

In order to quantify the selection pressure in coding regions of SARS-CoV-2, we employed dN/dS analysis, which assesses and compares non-synonymous to synonymous substitution rates. In its standard version, this analysis assumes uniform nucleotide substitution probabilities across the genome; however, this hypothesis might not hold if different mutational processes are active with biases over a subset of substitutions (e.g., non-uniform distribution might be observed across signature-based clusters). If this bias is not taken in account, it may lead to erroneous estimation of the dN/dS ratio (Van den Eynden and Larsson, 2017).

For this reason, since we discovered the existence of different host-related mutational processes (i.e., the mutational signatures) that are strongly biased toward specific substitutions, we developed a corrected-for-signatures dN/dS ratio analysis, as proposed in a different context in Van den Eynden and Larsson (2017). Specifically, given the ith sliding window of the coding region comprising l bases and considering the fth signature-based cluster, the corrected dN/dS ratio (for signature-cluster f and sliding window i) is given by:

$$\begin{aligned} &\text{corrected} \quad \frac{dN}{dS}^{i,f} = \frac{N_{obs}^{i,f}/S_{obs}^{i,f}}{\sum_{k=1}^{l}\sum_c P_{f,c}N_{k,c}/\sum_{k=1}^{l}\sum_c P_{f,c}S_{k,c}} \\ &c \in \{\mathsf{G{>}T{:}C{>}A}, \ldots, \mathsf{A{>}C{:}T{>}G}\} \quad \text{(6 substitution classes)} \end{aligned} \tag{1}$$

where, $N_{obs}^{i,f}$ and $S_{obs}^{i,f}$ are the numbers of non-synonymous and synonymous substitutions detected in the window i in at least one sample of signature-based cluster f, $P_{f,c}$ is the probability of substitution class c in signature-based cluster f (computed with respect to the categorical normalized cumulative VF distribution of all substitution classes in that cluster), and $N_{k,c}$ ($S_{k,c}$) is equal to 1 if class c identifies an admissible non-synonymous (synonymous) substitution in the kth position of the window i, 0 otherwise.

Phylogenetic model from clonal variant profiles via VERSO

VERSO is a 2-step computational framework for the characterization of viral evolution from raw sequencing data introduced in Ramazzotti et al. (2020). In particular, VERSO STEP #1 is a probabilistic noise-tolerant approach that processes binarized clonal variant profiles to deliver a robust phylogenetic model also in condition of sampling limitations and sequencing issues (for further details please refer to the related article).

In this work, we followed the guidelines proposed in Ramazzotti et al. (2020) and applied VERSO STEP #1 to the binarized mutational profiles of clonal mutations to reconstruct a phylogenetic model of the SARS-CoV-2 samples included in our dataset, and to investigate the possible presence of homoplasies of minor variants. In our analysis, we considered only clonal variants (VF $> 90\%$) detected in at least 3% of the samples of the dataset ($\tilde{m} = 28$ clonal variants on $n = 1133$ samples). The method was executed with 1 million MCMC iterations and returns 23 distinct clades associated to corrected clonal genotypes returned by the method. The stratification of samples in clades was employed in the analysis of homoplasies of minor variants.

We note that several widely used alternative approaches for phylogeny reconstruction from consensus sequences are available, among which, e.g., IQ-TREE (Nguyen et al., 2015), the algorithm included in the Nextstrain-Augur pipeline (Hadfield et al., 2018), BEAST 2 (Bouckaert et al., 2019) and MrBayes (Ronquist et al., 2012). In Supplementary Fig. S5 the phylogenetic model returned via MrBayes (Ronquist et al., 2012) on Dataset #1 is displayed (the method was executed with 10 million MCMC iterations and default parameters), showing consistent results with our analysis, as proven by the Adjusted Rand Index (ARI) (Santos and Embrechts, 2009) between sample partitionings (ARI = 0.76).

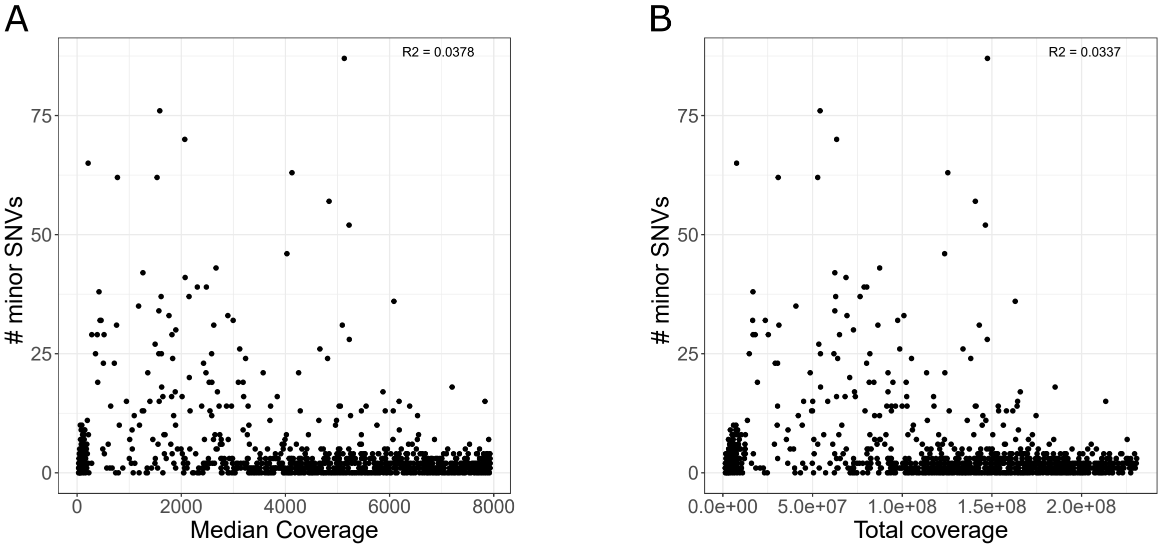

Figure S1: **Quality check – Dataset** #1 `PRJNA645906`. **Related to Figure** 1. (A) Scatter-plot returning for each sample of the cohort the number of minor SNVs (variant frequency VF $\leq 90\%$) and the median coverage. R^2 coefficient is shown in the upper-right corner. (B) Scatter-plot returning for each sample of the cohort the number of minor SNVs and the total coverage.

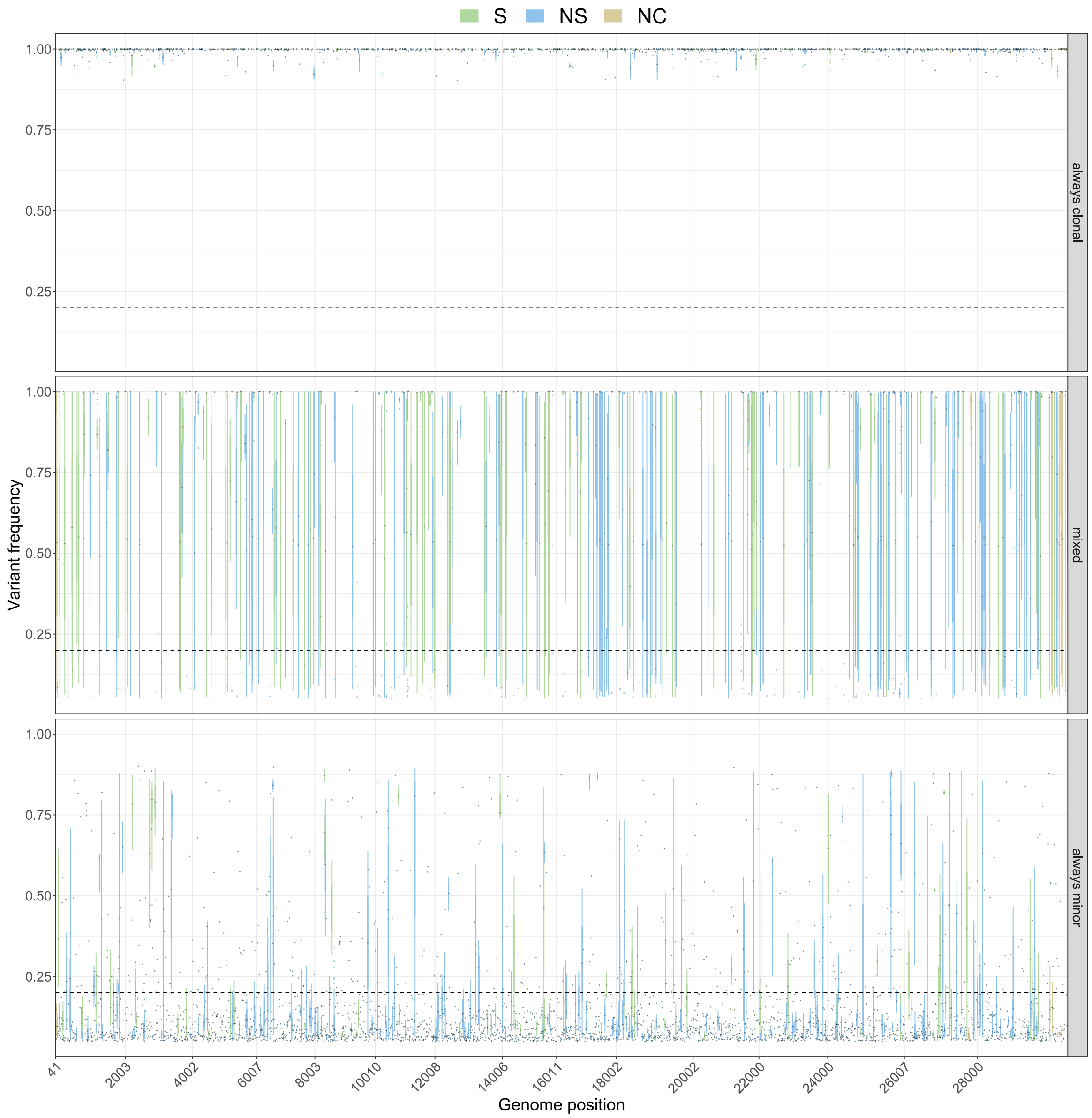

Figure S2: **Distribution of SNVs detected on SARS-CoV-2 genome – Dataset #1 `PRJNA645906`. Related to Figure** 2 Box-plots returning the VF distribution of all SNVs detected in all samples, colored according synonymous, non-synonymous and non-coding state and grouped according to SNV category.

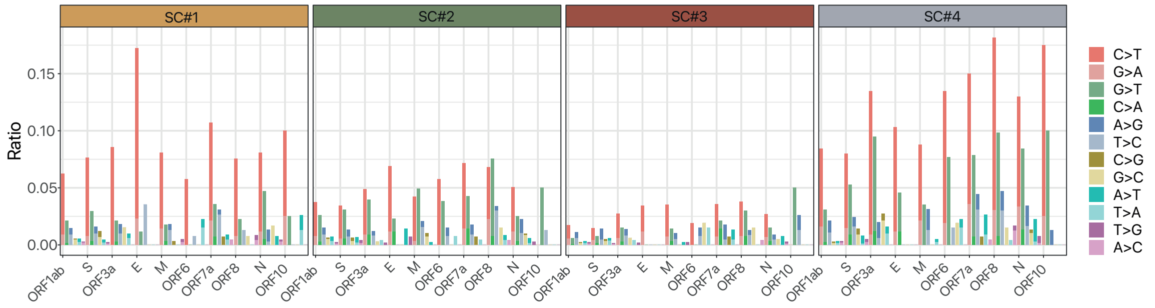

Figure S3: **Distribution of substitution types on SARS-CoV-2 ORFs – Dataset #1** `PRJNA645906`**. Related to Figure** 2. Barplots displaying the categorical distribution of all SNVs detected in all signature-based clusters, with respect SARS-CoV-2 ORFs (normalized by reference allele count in each ORF).

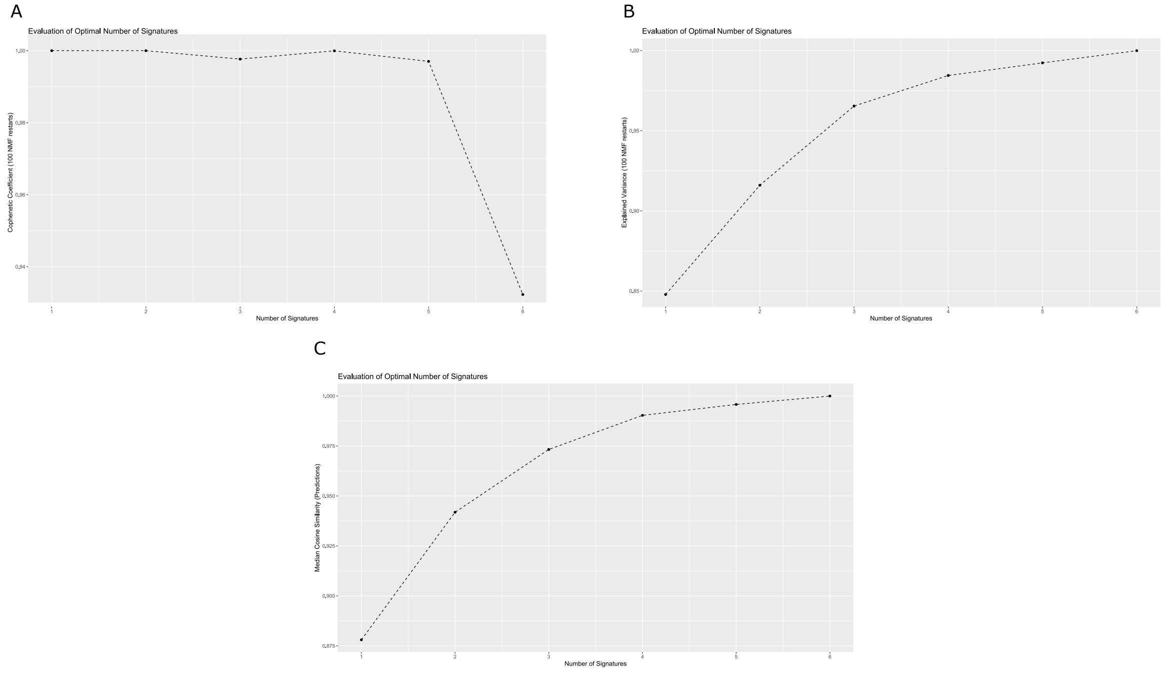

Figure S4: **Signature analysis: Cophenetic correlation coefficient, dispersion coefficient, explained variance, goodness of fit – Dataset #1** `PRJNA645906`**. Related to Figure** 3. (A) Explained variance, (B) average Cophenetic correlation coefficient with respect to Non-negative Matrix Factorization (NMF) rank, in the range 1 – 6. 1000 NMF restarts comprising 20 iterations were performed. A sharp drop is observed between 3 and 4, suggesting that the optimal rank (i.e., the number of signatures) is 3, (C) goodness of fit, measured as average Cosine correlation coefficient among observed profiles and predicted ones, with respect to NMF rank in the range 1 – 6 (Lal et al., 2020). A plateau is observed at rank 3. .

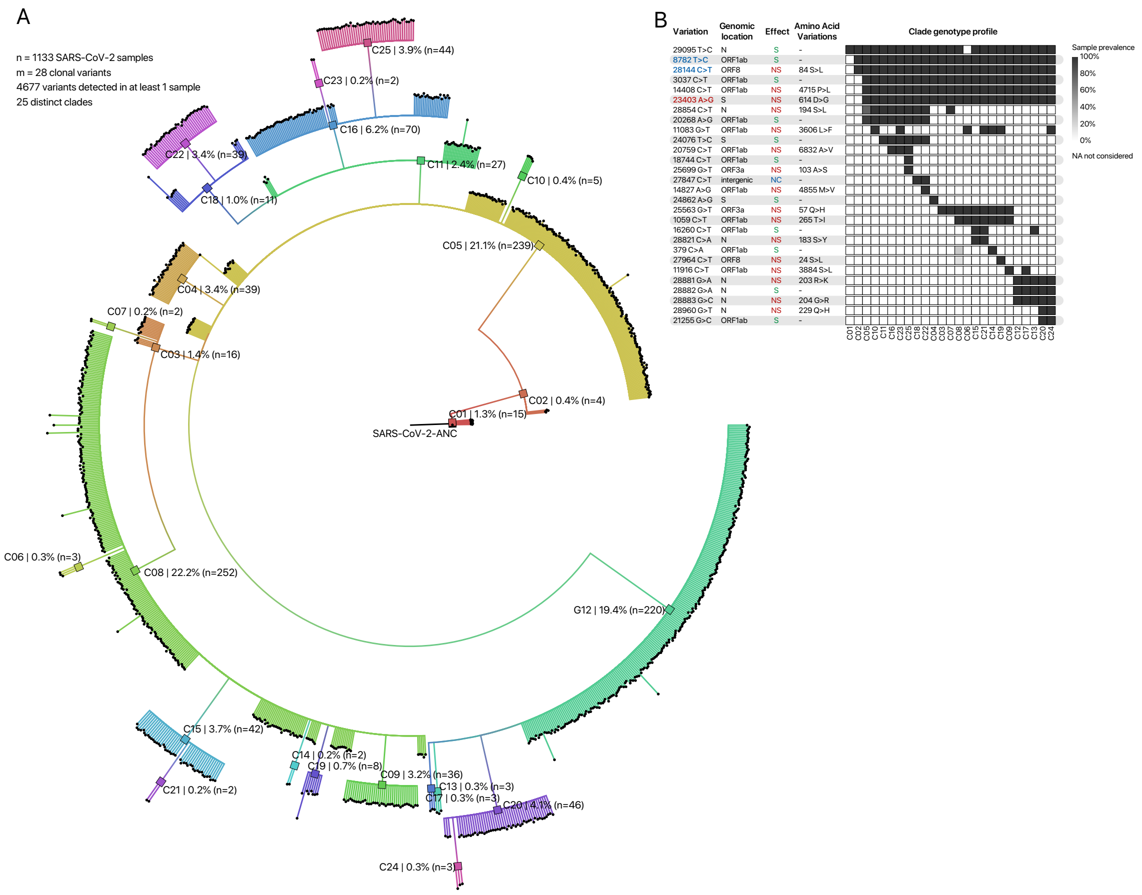

Figure S5: **Phylogenomic model of** 1133 **SARS-CoV-2 samples returned by MrBayes** – **Dataset** #1 PRJNA645906**. Related to Figure** 5. **(A)** The phylogenetic tree returned by MrBayes (Ronquist et al., 2012) considering 28 clonal variants (VF > 0.90) detected in at least 3% of the 1133 samples of the dataset is displayed. Colors mark the 25 clades identified by MrBayes (visualization via FigTree (Rambaut, 2009)). The black colored sample represents the SARS-CoV-2-ANC reference genome. **(B)** Heatmap returning the fraction of samples of any clade in which a specific clonal variant is observed. Clonal SNVs are annotated with mapping on ORFs, synonymous (S), nonsynonymous (NS) and non-coding (NC) states, and related amino acid substitutions. Variants g.8782T>C (*ORF1ab*, synonymous) and g.28144C>T (*ORF8*, p.84S>L) are colored in blue, whereas variant g.23403 A>G (*S*, p.614 D>G) is colored in red. The results are consistent with those obtained by VERSO STEP #1 (Figure 5 of the main text), as proven by the Adjusted Rand Index (ARI) (Santos and Embrechts, 2009) between sample partitionings (ARI $= 0.76$)

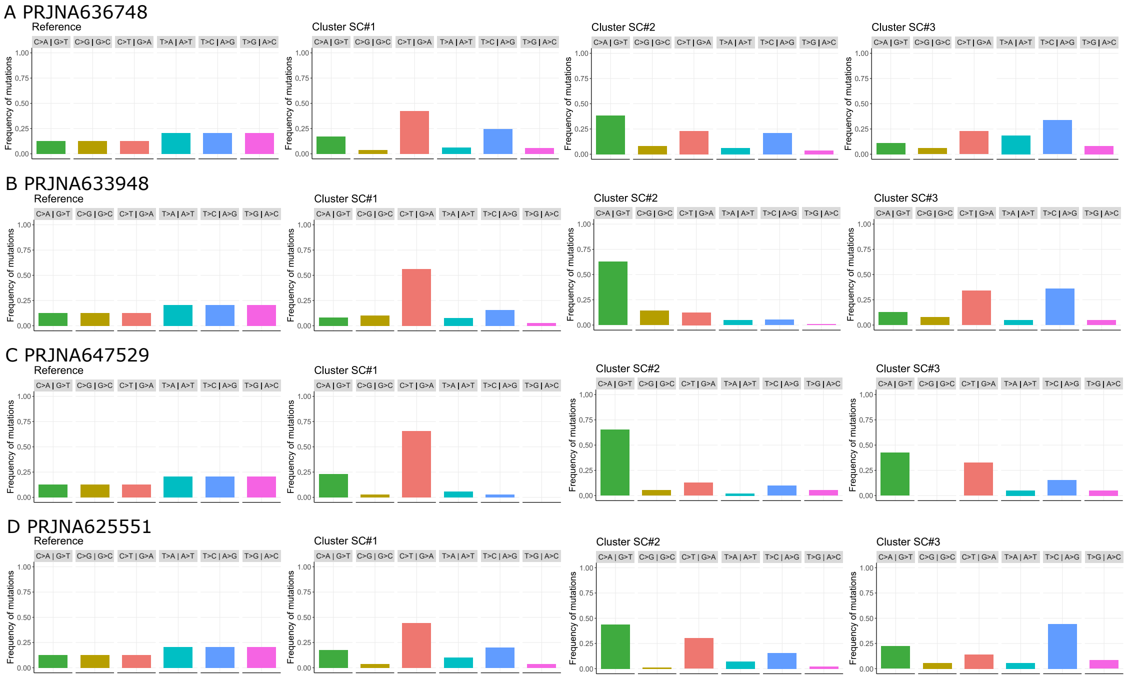

Figure S6: **Categorical substitution type distribution – Validation datasets** $\#2, 3, 4, 5$ **(**`PRJNA: 636748, 633948, 647529, 625551`**). Related to Figure** 6. Categorical normalized cumulative distribution of all SNVs detected in each signature-based cluster of the validation datasets, with respect to 6 substitution classes.

References

Alexandrov, L.B., Nik-Zainal, S., Wedge, D.C., Aparicio, S.A., Behjati, S., Biankin, A.V., Bignell, G.R., Bolli, N., Borg, A., Børresen-Dale, A.L., et al. (2013). Signatures of mutational processes in human cancer. Nature 500, 415–421.

Andersen, K.G., Rambaut, A., Lipkin, W.I., Holmes, E.C., and Garry, R.F. (2020). The proximal origin of SARS-CoV-2. Nat. Med. 26, 450–452.

Bastola, A., Sah, R., Rodriguez-Morales, A.J., Lal, B.K., Jha, R., Ojha, H.C., Shrestha, B., Chu, D.K., Poon, L.L., Costello, A., et al. (2020). The first 2019 novel coronavirus case in Nepal. Lancet Infect Dis 20, 279–280.

Bouckaert, R., Vaughan, T.G., Barido-Sottani, J., Duchêne, S., Fourment, M., Gavryushkina, A., Heled, J., Jones, G., Kühnert, D., De Maio, N., et al. (2019). Beast 2.5: An advanced software platform for bayesian evolutionary analysis. PLoS Comput. Biol 15, e1006650.

Brunet, J.P., Tamayo, P., Golub, T.R., and Mesirov, J.P. (2004). Metagenes and molecular pattern discovery using matrix factorization. Proc. Natl. Acad. Sci. USA 101, 4164–4169.

Chen, D. and Plemmons, R.J. (2010). Nonnegativity constraints in numerical analysis. In The birth of numerical analysis, pages 109–139 (World Scientific).

Hadfield, J., Megill, C., Bell, S.M., Huddleston, J., Potter, B., Callender, C., Sagulenko, P., Bedford, T., and Neher, R.A. (2018). Nextstrain: real-time tracking of pathogen evolution. Bioinformatics 34, 4121–4123.

Koboldt, D.C., Zhang, Q., Larson, D.E., Shen, D., McLellan, M.D., Lin, L., Miller, C.A., Mardis, E.R., Ding, L., and Wilson, R.K. (2012). VarScan 2: somatic mutation and copy number alteration discovery in cancer by exome sequencing. Genome Res. 22, 568–576.

Lal, A., Liu, K., Tibshirani, R., Sidow, A., and Ramazzotti, D. (2020). De novo mutational signature discovery in tumor genomes using sparsesignatures. bioRxiv. https://doi.org/10.1101/384834.

Nguyen, L.T., Schmidt, H.A., Von Haeseler, A., and Minh, B.Q. (2015). IQ-TREE: a fast and effective stochastic algorithm for estimating maximum-likelihood phylogenies. Mol Biol Evo 32, 268–274.

Ramazzotti, D., Angaroni, F., Maspero, D., Gambacorti-Passerini, C., Antoniotti, M., Graudenzi, A., and Piazza, R. (2020). VERSO: a comprehensive framework for the inference of robust phylogenies and the quantification of intra-host genomic diversity of viral samples. bioRxiv. https://doi.org/10.1101/2020.04.22.044404.

Rambaut, A. (2009). Figtree v1. 3.1. http://tree. bio. ed. ac. uk/software/figtree/ .

Ronquist, F., Teslenko, M., van der Mark, P., Ayres, D.L., Darling, A., Höhna, S., Larget, B., Liu, L., Suchard, M.A., and Huelsenbeck, J.P. (2012). MrBayes 3.2: Efficient Bayesian Phylogenetic Inference and Model Choice Across a Large Model Space. Syst. Biol 61, 539–542.

Santos, J.M. and Embrechts, M. (2009). On the use of the Adjusted Rand Index as a metric for evaluating supervised classification. In C. Alippi, M. Polycarpou, C. Panayiotou, and G. Ellinas (Eds.), Artificial Neural Networks – ICANN 2009, pages 175–184 (Springer Berlin Heidelberg).

Van den Eynden, J. and Larsson, E. (2017). Mutational signatures are critical for proper estimation of purifying selection pressures in cancer somatic mutation data when using the dN/dS metric. Front. Genet. 8, 74.

Wilson, D.J. (2019). The harmonic mean p-value for combining dependent tests. Proc. Natl. Acad. Sci. USA 116, 1195–1200.

Xiao, K., Zhai, J., Feng, Y., Zhou, N., Zhang, X., Zou, J.J., Li, N., Guo, Y., Li, X., Shen, X., et al. (2020). Isolation of SARS-CoV-2-related coronavirus from malayan pangolins. Nature 583, 286–289.

Zhou, P., Yang, X.L., Wang, X.G., Hu, B., Zhang, L., Zhang, W., Si, H.R., Zhu, Y., Li, B., Huang, C.L., et al. (2020). A pneumonia outbreak associated with a new coronavirus of probable bat origin. Nature 579, 270–273.
